# Revealing sRNA expression profiles of NDM-5-producing CRKP and explore the role of sRNA207 in NDM-5-producing CRKP resistance

**DOI:** 10.1128/spectrum.01537-24

**Published:** 2024-11-07

**Authors:** Chonghong Xie, Yibo Bai, Yan Li, Bing Cui, Guixue Cheng, Jianhua Liu, Yong liu, Xiaosong Qin

**Affiliations:** 1 Department of Laboratory Medicine, Shengjing Hospital of China Medical University, Shenyang, Liaoning, China; 2 Liaoning Clinical Research Center for Laboratory Medicine, Shenyang, Liaoning, China; 3 Ziyang College of Dental Technology, Ziyang, Sichuan, China; Forschungszentrum Jülich GmbH, Juelich, Germany

**Keywords:** microbiology, antibiotic resistance, *Klebsiella*

## Abstract

New Delhi metallo-beta-lactamase-5 (NDM-5)-producing carbapenem-resistant *Klebsiella pneumoniae* (CRKP) is characterized by high virulence, high morbidity, and mortality, and the detection rate in children has increased in recent years. Therefore, it is urgent to develop new therapeutic targets and strategies. Non-coding small RNA (sRNA)-mediated RNA-based therapies offer a new direction for the treatment of bacterial infections, especially resistant bacteria. This study first analyzed the transcriptional expression profiles of NDM-5-producing CRKP and Carbapenem-susceptible *Klebsiella pneumoniae* (CSKP) isolates from the clinic by RNA-seq. A total of 4,623 genes were obtained, of which 307 genes were differentially expressed in NDM-5-producing CRKP, and these differentially expressed genes are mainly related to metabolism. Then, by analyzing the length and secondary structure of genes that could not match the reference gene and non-redundant protein database, we obtained 268 sRNAs, of which 13 sRNAs were differentially expressed in NDM-5-producing CRKP. After the expression level of differentially expressed sRNA was verified by RT-PCR to be consistent with that of RNA-seq, we chose sRNA207 as our research target. By knockdown of sRNA207 and *smf-1* (the predicted target mRNA of sRNA207) in the strain, we found that increased expression of sRNA207 promoted biofilm formation by stabilizing expression of *smf*-1, which in turn affected the resistance of NDM-5-producing CRKP to carbapenems. This provides a new approach to treat CRKP infection.

**IMPORTANCE:**

sRNAs form a regulatory network that regulates bacterial virulence, drug resistance, and other functions by targeting mRNAs. However, sRNA expression profile and function of NDM-5-producing carbapenem-resistant *Klebsiella pneumoniae* (CRKP) are still unknown. In this study, we analyzed the sRNA expression profiles of NDM-5-producing CRKP obtained from clinical by referring to the methods of previous articles. A total of 268 candidates sRNAs were obtained, of which 248 were newly discovered. More importantly, 13 sRNAs were differentially expressed in NDM-5-producing CRKP compared with CSKP. We knocked down sRNA207 in NDM-5-producing CRKP to validate its effect on *smf*-1, biofilm, and resistance of strains. We also confirmed the role of *smf*-1 in biofilm formation and drug resistance of NDM-5-producing CRKP by constructing *smf*-1-knockdown strain. The results suggest that *smf*-1 is the target gene of sRNA207. Increased expression of sRNA207 in NDM-5-producing CRKP stabilizes *smf*-1 expression, which in turn affects the resistance of the strains through biofilm formation.

## INTRODUCTION

*Klebsiella pneumoniae* is one of the most important pathogens of community-acquired and nosocomial infections, which can cause respiratory, urinary, and even blood infections (1). Its problem of drug resistance, particularly carbapenemase resistance, is a particularly difficult challenge for clinical management and infection control (2).

Carbapenemase-resistant *K. pneumoniae* (CRKP) can be divided into various types according to the production of different carbapenem enzymes, and it is worth noting that the detection rate for NDM-producing CRKP has risen in recent years (3, 4). NDM-producing CRKP has been listed as an “urgent threat“ by the US Centers for Disease Control and Prevention in 2019 (2). NDM carbapenemases are characterized by rapid and continuous evolution and are divided into NDM-1 to NDM-24 (5, 6). Among them, NDM-1 and NDM-5 types are commonly detected (3). In contrast to NDM-1, NDM-5 is a two amino acids variant (7). This mutation caused NDM-5 to have a higher hydrolytic activity than NDM-1, and greater carbapenem resistance than other metallo-beta-lactamases (8, 9). More importantly, NDM-5-producing CRKP infection in children and newborns has been increasingly reported (3, 6, 10, 11). Due to the high resistance level of NDM-5-producing CRKP and the limited availability of effective treatments, new treatment strategies are urgently needed.

Non-coding RNA-mediated RNA-based therapies have been successfully used in the treatment of various diseases, including cancer (12). In recent years, more and more attention has been paid to its application in antibacterial therapy (13). It is well known that bacteria can develop resistance to antibiotics by regulating the expression, stability, and translation efficiency of RNA (14, 15). For example, *Pseudomonas aeruginosa* is resistant to imipenem by downregulating the expression of oprD (16). In addition, the increased expression of pbp5 mRNA resulted in ampicillin resistance in *Enterococcus faecium* (17). Recent studies have found that a non-coding RNA (sRNA), with a length of 50–500 nt, forms a regulatory network by targeting mRNAs to regulate bacterial virulence, drug resistance, and other functions (18–20). sRNA PA0805.1 appeared to be a global regulator that influences motility, mytotoxicity, and tobramycin resistance of *P. aeruginosa* (21). In our previous studies, we found that sRNA51 regulates KPC-2-producing CRKP carbapenems resistance by inhibiting the expression of *acrA* (22). There have been studies to explore the application of synthetic sRNAs in bacterial metabolism and biology (14, 23). However, the sRNA transcription expression profile and the function of sRNA in NDM-5-producing CRKP are still unknown.

Bacteria can form biofilms against harsh environmental conditions, including antibiotics (24). Biofilm plays an important role in bacterial infection and drug resistance (25, 26). In recent years, the correlation between antibiotic resistance in *K. pneumoniae* and biofilm formation ability has also been demonstrated, on the one hand, multidrug-resistant *K. pneumoniae* (including CRKP) has stronger biofilm formation ability (27, 28); on the other hand, antibiotic resistance of *K. pneumoniae* in the biofilm state increased compared with free-living form (29). Studies have found that NDM-5-producing *Escherichia coli* biofilm formation ability is enhanced (30). However, the biofilm-generating capacity of NDM-5-producing CRKP and the role of biofilm in its drug resistance are still unclear.

Therefore, we aimed to use RNA-seq to reveal the sRNA expression profiles of NDM-5-producing CRKP. Then, by knocking down the expression of sRNA207 and *smf-1* in NDM-5-producing CRKP, we explored the mechanism by which sRNA207 affects the resistance of NDM-5-producing CRKP to carbapenem antibiotics by affecting biofilm formation, providing a new therapeutic target for clinical application.

## RESULTS

### Detection resistance of CSKP and NDM-5-producing CRKP to common antibiotics

We randomly selected eight CSKP strains and eight NDM-5-producing CRKP strains from the strain bank of Shengjing Hospital of China Medical University. The specific information about these strains is shown in Table S1. At first, we verified their resistance using the Phoenix-100 compact system, and the results showed that these NDM-5-producing CRKP strains were resistant to carbapenems and other drugs such as piperacillin/tazobactam and amoxicillin/clavulanic acid. All strains were also resistant to ampicillin, and studies have shown that *K. pneumoniae* is naturally resistant to it (31) (Table 1). These results are consistent with previous studies (31, 32). Because we were particularly concerned about the level of resistance to carbapenems of these strains, we further measured the minimum inhibitory concentration (MIC) of meropenem, ertapenem, and imipenem. The results are shown in Table 1.

**TABLE 1:** Antibiotic resistance and MICs of carbapenems of *Klebsiella pneumoniae*^*a*^

	CSKP								NDM-5 CRKP							
	1	2	3	4	5	6	7	8	1	2	3	4	5	6	7	8
Ceftriaxone	S	S	S	S	S	S	S	S	R	R	R	R	R	R	R	R
Ceftazidime	S	S	S	S	S	S	S	S	R	R	R	R	R	R	R	R
Cefoperazone/sulbactam	S	S	S	S	S	S	S	S	R	R	R	R	R	R	R	R
Piperacillin/tazobactam	S	S	S	S	S	S	S	S	R	R	R	R	R	R	R	R
Amoxicillin/clavulanic acid	S	S	S	S	S	S	S	S	R	R	R	R	R	R	R	R
Cefepime	S	S	S	S	S	S	S	S	R	R	R	R	R	R	R	R
Cefoxitin	S	S	S	S	S	S	S	S	R	R	R	R	R	R	R	R
Ampicillin^*b*^	R	R	R	R	R	R	R	R	R	R	R	R	R	R	R	R
Cefazolin	S	S	S	S	S	S	S	S	R	R	R	R	R	R	R	R
Tetracycline	S	S	S	S	S	S	S	S	R	R	S	R	S	R	R	S
Meropenem (MIC, μg/mL)	S (≤1)	S (≤1)	S (≤1)	S (≤1)	S (≤1)	S (≤1)	S (≤1)	S (≤1)	R (64)	R (64)	R (128)	R (64)	R (64)	R (128)	R (128)	R (128)
Ertapenem (MIC, μg/mL)	S (≤1)	S (≤1)	S (≤1)	S (≤1)	S (≤1)	S (≤1)	S (≤1)	S (≤1)	R (64)	R (128)	R (128)	R (64)	R (128)	R (128)	R (128)	R (128)
Imipenem (MIC, μg/mL)	S (≤1)	S (≤1)	S (≤1)	S (≤1)	S (≤1)	S (≤1)	S (≤1)	S (≤1)	R (64)	R (128)	R (128)	R (64)	R (128)	R (64)	R (128)	R (128)

^*a*^ CSKP was from pneumonia patients, and NDM-5-producing CRKP was from septicemia children. “S” stands for drug sensitivity, and “R” stands for drug resistance.

^*b*^ *K. pneumoniae* is naturally resistant to ampicillin (31).

### Transcription expression profile obtaining and differentially expressed genes analysis of NDM-5-producing CRKP

**Fig 1:**
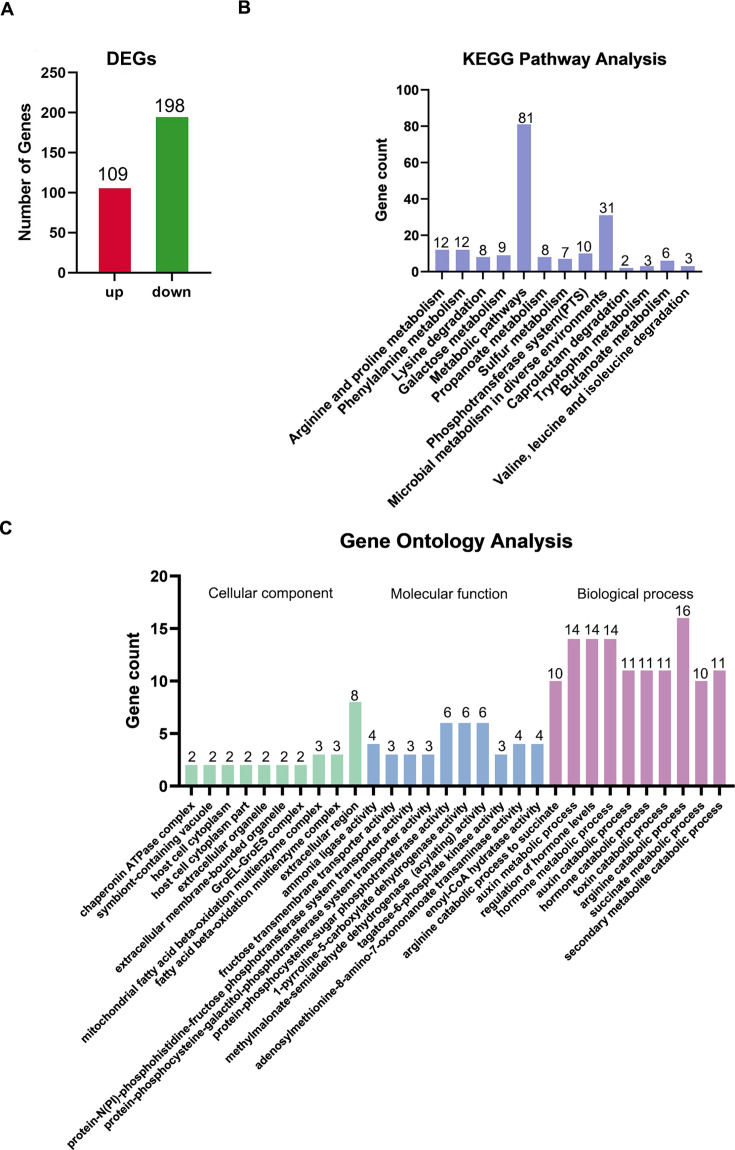
Screening and functional analysis of DEGs in NDM-5-producing CRKP. (**A**) FDR and log_2_FC were used to screen DEGs. (**B**) Kyoto Encyclopedia of Genes and Genomes pathway analysis of DEGs. There represents 13 significantly (*P* < 0.05) genes pathway enrichment. (**C**) Gene ontology classification. DEGs were annotated into three categories: biological processes, molecular functions, and cellular components. *Y*-axis represents the number of genes corresponding to each classification entry. *X*-axis represents meaningful (*P* < 0.05) enrichment pathway items.

We randomly selected four NDM-5-producing CRKP and four CSKP strains from the above 16 bacteria for RNA-seq and obtained 4,623 genes by comparing RNA-seq reads with the reference genome assembly GCF022869665.1 through Bowtie2 software (approximately 82%–89% of the reads were mapped). Data generated were deposited in the Sequence Read Archive (SRA) at the National Center for Biotechnology Institute (accession no. PRJNA1006790). In addition, with the criteria of false discovery rate (FDR) <0.05 and |log_2_FC| > 1, we obtained 307 differentially expressed genes (DEGs) in NDM-5-producing CRKP compared to CSKP, including 109 upregulated and 198 downregulated genes (Fig. 1A; Table S2).

To understand the function of these DEGs, we performed Kyoto Encyclopedia of Genes and Genomes (KEGG) pathway enrichment and gene ontology (GO) analysis. The KEGG pathway analysis results showed that DEGs were mapped to 73 KEGG pathways (Table S3), 13 of which were significantly enriched (*P* < 0.05) (Fig. 1B). Moreover, GO classification results suggested that DEGs significantly enriched (*P* < 0.05) 24 terms in cellular components, 32 terms in molecular functions, and 81 terms in biological processes (Table S4), with the top 10 items significantly enriched in each class were exhibited in Fig. 1C. And both the results of KEGG pathway analysis and GO classification showed that these DEGs are mainly related to metabolism.

### Revealing the sRNA expression profile and the differentially expressed sRNA of NDM-5-producing CRKP

By analyzing the length and secondary structure of unannotated transcripts, which are gained by comparing RNA-seq reads with the reference genome and non-redundant protein (NR) database, we obtained 268 candidate sRNAs, with length from 50 to 500 nt. Comparing with sRNAMap and Rfam databases, we found that 248 of them were uncovered sRNAs (Table S5). Of candidate sRNAs, 13 sRNAs were differentially expressed in NDM-5-producing CRKP with the criteria of FDR < 0.05 and |log_2_FC| > 1, including 3 upregulated and 10 downregulated sRNAs (Table 2).

**TABLE 2:** Significant differences in sRNA in NDM-5-producing CRKP^*a*^

Gene	log_2_(FC)	CSKP FPKM	NDM-5 FPKM	*P* value	FDR	Length (nt)
sRNA207	16.43	0.0010	88.1450	0.0000	0.0001	136
sRNA497	4.85	4.0233	116.3400	0.0012	0.0288	137
sRNA32	4.59	5.0233	121.3550	0.0001	0.0034	178
sRNA134	−3.72	480.2300	36.3525	0.0001	0.0034	84
sRNA77	−3.99	105.1667	6.6075	0.0008	0.0205	132
sRNA132	−4.02	214.7067	13.2100	0.0001	0.0034	68
sRNA155	−4.56	133.8200	5.6650	0.0018	0.0398	65
sRNA93	−13.35	1045.0470	0.1000	0.0000	0.0000	98
sRNA62	−14.73	27.100	0.0010	0.0003	0.0093	60
sRNA33	−15.62	50.2100	0.0010	0.0000	0.0000	52
sRNA14	−15.83	58.4467	0.0010	0.0001	0.0031	74
sRNA316	−16.66	103.7100	0.0010	0.0000	0.0005	166
sRNA504	−17.77	223.1300	0.0010	0.0000	0.0017	70

^*a*^ The expression level of sRNA was calculated per million base pair transcripts (FPKM). Log_2_(FC) represents the ratio of expression between the two groups and was logarithmic, with a base of two. *P* value indicates the statistical test value, FDR is the false discovery rate and obtained by correcting for the *P* value; both FDR and *P* < 0.05 represent statistically significant differences. Length (nt) represents the length of the sRNA.

### Validating the expression level of differentially expressed sRNAs in NDM-5-producing CRKP

**Fig 2:**
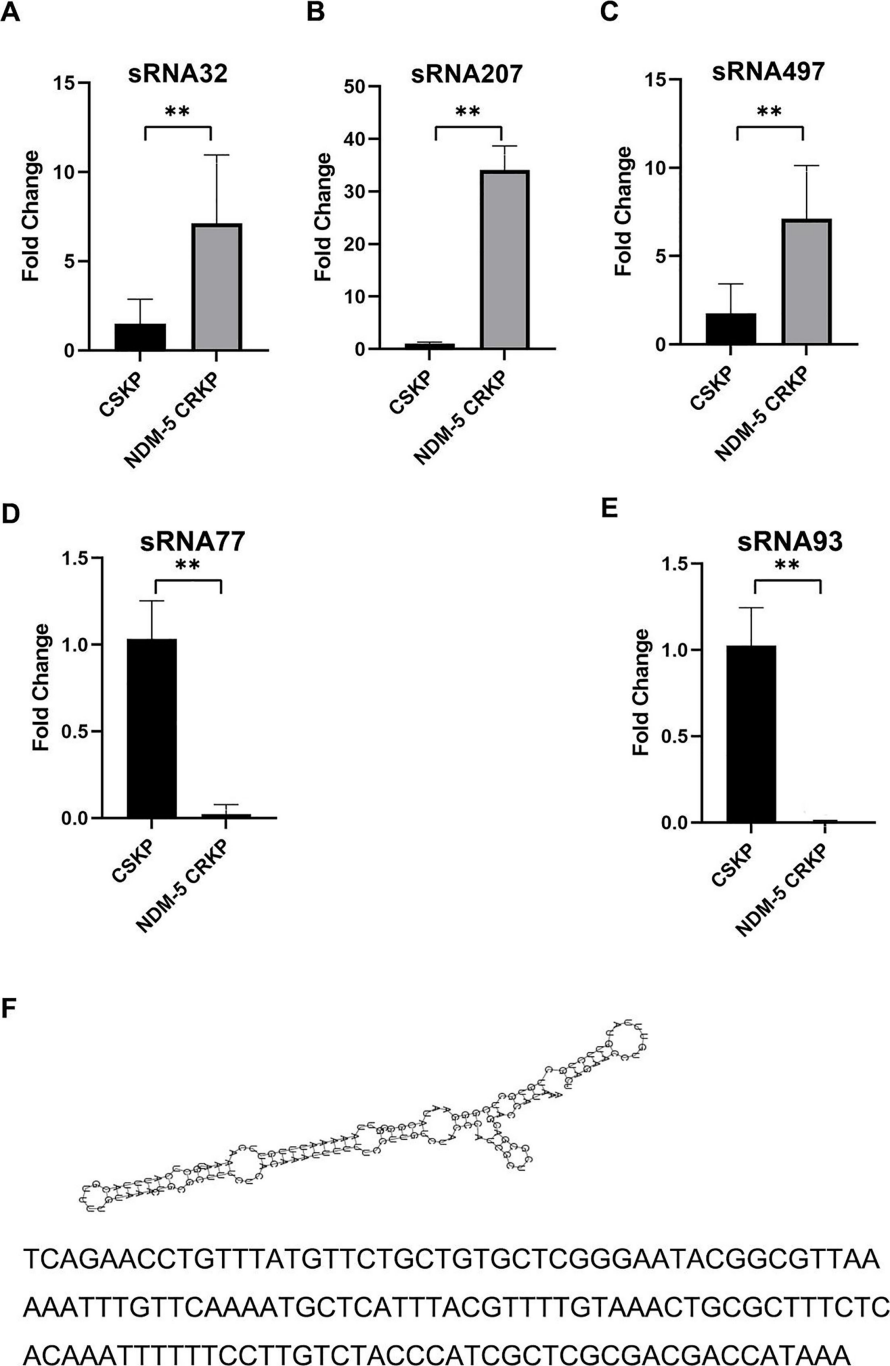
Validation and analysis of differentially expressed sRNAs in NDM-5-producing CRKP. Expression of (**A**) sRNA32, (**B**) sRNA207, (**C**) sRNA497, (**D**) sRNA77, and (**E**) sRNA93 in CSKP, and NDM-5-producing CRKP was detected and compared by qRT-PCR. The data were expressed as mean ± SD (*n* = 8), **P < 0.01. (**F**) Secondary structure and gene sequence of sRNA207 were analyzed by RNAfold software.

To validate the presence and expression level of differentially expressed sRNAs obtained by RNA-seq, we randomly selected three upregulated (sRNA32, sRNA207, sRNA497) and two downregulated (sRNA77 and sRNA93) to examine the transcript levels by Quantitative Real-time PCR (qRT-PCR), and the results indicated that the expression level of these sRNAs was matched those of the RNA-seq (Fig. 2A through E). Furthermore, among the above five sRNAs, RNA-seq and qRT-PCR results showed that the expression of sRNA207 was the most significantly differential in NDM-5-producing CRKP, 16-fold and 34-fold higher than that in CSKP, respectively. Therefore, we chose sRNA207 for further study and mined its gene sequence, secondary structure, and length as shown in Fig. 2F. The study has found that protein-coding RNAs all have open reading, while sRNAs are intergenic regions between open reading frames (ORFs), and therefore, do not have the ability to encode proteins (33). So, we used the CPAT software to detect whether sRNA207 had ORF to determine whether sRNA207 had protein-coding function. The results showed that the sRNA207 Fickett score <0.74 and Hexamer score had negative values, indicating that it did not have ORF and protein-coding capacity (34, 35).

### Prediction and validation of the target mRNAs of sRNA207

**Fig 3:**
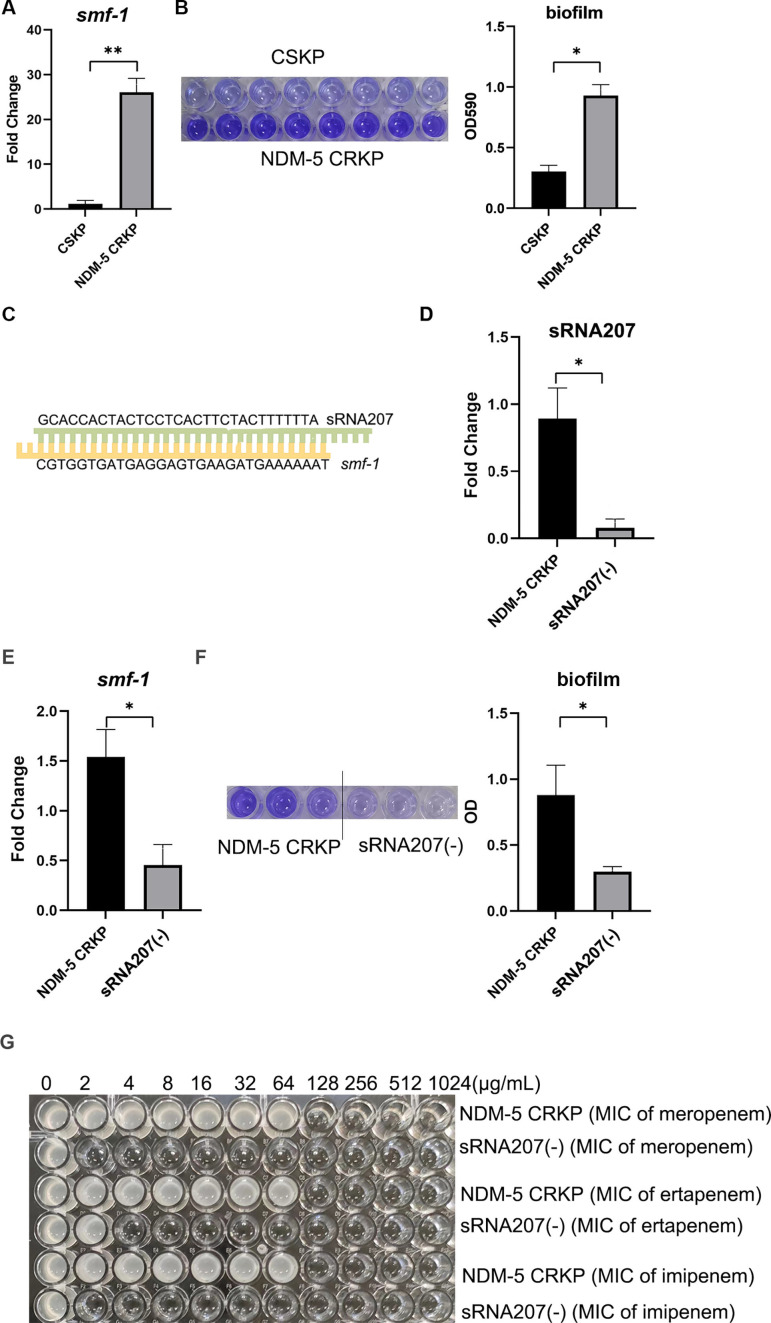
Functional validation of sRNA207. (**A**) Expression levels of *smf-1* in CSKP and NDM-5-producing CRKP were detected by qRT-PCR. (**B**) The biofilm formation of CSKP and NDM-5-producing CRKP was investigated by crystal violet staining, and quantification data of biofilm formation were shown as histogram. (**C**) A schematic representation of the binding of sRNA207 and *smf-1* which is predicted by IntaRNA 2.0.3. The data were expressed as mean ± SD (*n* = 8), **P < 0.005. qRT-PCR was used to validate the expression of (**D**) sRNA207 and (**E**) *smf-1* in NDM-5-producing CRKP and sRNA207(−) strain (*n* = 3). (**F**) The biofilm formation of NDM-5-producing CRKP and sRNA207(−) strain was detected by crystal violet staining (*n* = 3). Quantification data of biofilm formation were shown as histogram. (**G**) Resistance of NDM-5-producing CRKP and sRNA207(−) strains to meropenem, ertapenem, and imipenem was detected by microbroth dilution. Data are represented as the mean ± SD (*n* = 3), *P < 0.05.

To explore the role of sRNA207 in NDM-5-producing CRKP, we used IntaRNA 2.0.3 to predict the target mRNAs of it, which were shown to include *yciG*, *glpR*, *ycgZ*, *smf-1*, *deoA*, and *ilvG* (Table S6). Among them, *smf-1* has attracted our attention, because studies have found that *smf-1* can promote the formation of biofilm (36, 37). As we all know, the biofilm can cause and enhance the resistance of *K. pneumoniae* (38). Through qRT-PCR and biofilm experiments, we found that the *smf-1* expression level and biofilm formation ability of NDM-5-producing CRKP were higher than that of CSKP (Fig. 3A and B). In addition, we predicted that sRNA207 can bind to the coding region of *smf-1* and drew a simple diagram, as shown in Fig. 3C.

To confirm the effects of sRNA207 on *smf-1* expression and biofilm formation, we knocked down sRNA207 in NDM-5-producing CRKP and found that the expression of *smf-1* and biofilm formation decreased in sRNA207(−) strain compared to NDM-5-producing CRKP (Fig. 3E and F). Subsequently, we also tested the carbapenems resistance of sRNA207(−) strains and found that the sRNA207(−) strains showed reduced resistance to meropenem from 128 μg/mL to 2 μg/mL, ertapenem from 128 μg/mL to 4 μg/mL, and imipenem 128 µg/mL to <2 µg/mL (Fig. 3G).

### sRNA207 regulates the resistance of NDM-5-producing CRKP through *smf-1*

**Fig 4:**
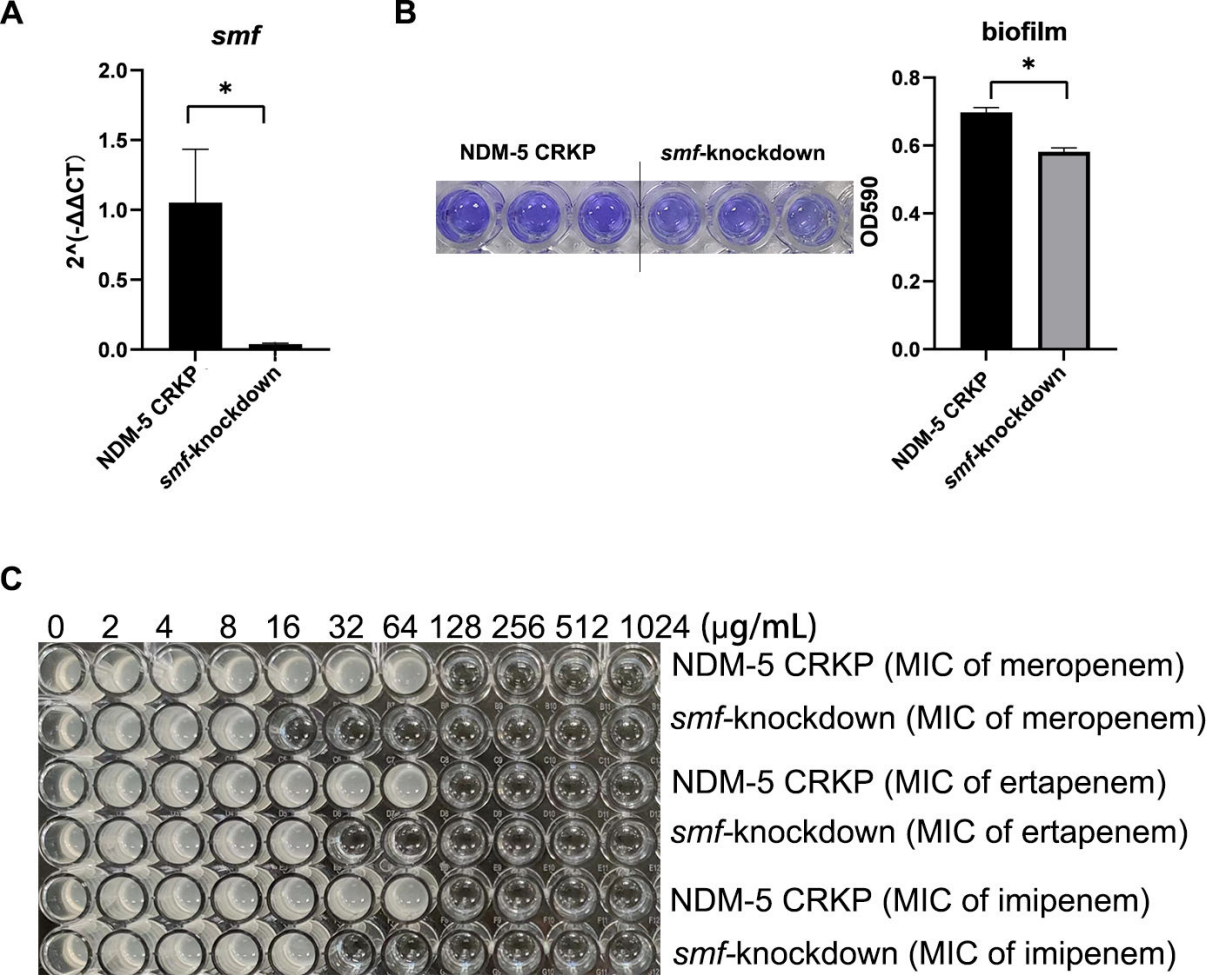
Mechanism of sRNA207 regulation carbapenems resistance of NDM-5-producing CRKP. qRT-PCR was used to validate the expression of (**A**) *smf-1* in NDM-5 producing CRKP and *smf*-knockdown strain. (**B**) The biofilm formation of NDM-5-producing CRKP and *smf*-knockdown strain was detected by crystal violet staining. Quantification data of biofilm formation were shown as histogram. (**C**) Resistance of NDM-5-producing CRKP and *smf*-knockdown strains to meropenem, ertapenem, and imipenem was detected by microbroth dilution. Data are represented as the mean ± SD (*n* = 3), *P < 0.05.

Based on the above results, we hypothesized that sRNA207 affects biofilm formation by regulating the expression of *smf-1*, and thus affects the resistance of NDM-5-producing CRKP. To confirm this hypothesis, we constructed *smf-1*-knockdown strains. The results of biofilm experiments and minimum inhibitory concentration measuring showed that, compared with NDM-5-producing CRKP, the biofilm of *smf-1*-knockdown strains was reduced (Fig. 4B), and the MIC of s*mf-1*-knockdown strains decreased from 128 μg/mL to 16 μg/mL for meropenem, from 128 μg/mL to 32 μg/mL for ertapenem, and from 128 μg/mL to 32 μg/mL for imipenem (Fig. 4C).

## DISCUSSION

In recent years, the detection rate of NDM-5-produing CRKP has gradually increased, especially in children (10, 39). Of 50 carbapenem-resistant *Klebsiella aerogenes* isolates collected from children in a pediatric hospital of Shanghai in 2019, 45 strains produced NDM-5, attention should be paid to control the prevalence of NDM-5 in children (3). Such concerns are necessary, because NDM-5 has a higher resistance, and children’s immune system development is not perfect, resulting in limited drug use, which brings great challenges for clinical treatment (10). Therefore, new therapeutic strategies and targets are urgently needed.

The good clinical efficacy of ceftazidime/avibactam in the treatment of KPC-2-producing CRKP infections suggests that re-sensitizing-resistant strains to existing antibiotics is a promising strategy (40). Unfortunately, ceftazidime/avibactam has no activity against NDM-5 (41, 42). The discovery of sRNA in bacteria has led to new possible strategies for researchers. sRNA, which is considered as an RNA-based therapeutic target, similar to microRNA in eukaryotes, does not have the function of encoding proteins but can stabilize or inhibit mRNAs expression by binding to them, thus affecting various functions of bacteria (43–45). sRNA EsrF directly interacts with the flagellar biosynthetic gene, *flhB*, increase its abundance, thereby leading to elevated *Escherichia coli O157:H7* motility and virulence (46). In *Shigella sonnei*, sRNA1039 regulates the expression of pore protein gene *ompD* thereby regulating bacterial sensitivity to ampicillin, gentamicin, and cefuroxime (47).

To obtain the sRNA expression profile of the NDM-5-producing CRKP, we first analyzed the transcription expression profile of eight clinical isolates using RNA-seq. RNA-seq is a commonly used and effective tool for mining bacterial genetic information. Qin N et al. used RNA-Seq to investigate the regulatory mechanism responsible for the inhibitory effect of sodium propionate on methicillin-resistant *Staphylococcus aureus* (48). Miyakoshi et al. used RNA-Seq to identify more novel target genes of sRNA GcvB in *Escherichia coli* (49). After aligning the RNA-seq readings to the reference genome, we obtained 4,623 genes including 307 DEGs in NDM-5-producing CRKP. Similar to Su Min Kyung’s findings, these differential genes were implicated in antibiotic target alterations, which were associated with resistance to drugs such as quinolones and tetracyclines (50). To further understand the function of DEGs, we performed KEGG pathway enrichment and GO analysis. KEGG pathway enrichment analysis showed that the DEGs were related to arginine and proline metabolism, L-phenylalanine metabolism, lysine degradation, and tryptophan metabolism. Furthermore, GO analysis showed that the DEGs were mainly related to protein complex, oxidoreductase activity, and various metabolic functions. Therefore, these DEGs are mainly involved in metabolic processes. It has been found that metabolism plays an important role in bacterial growth, drug resistance, and pathogenesis (51). For example, a shift in the glucose metabolism pathway in *E. coli* activates the toxin-antitoxin system of the bacteria, thereby developing resistance to fluoroquinolones (52). Bernier et al. found that the addition of amino acids such as L-phenylalanine, ornithine, and tyrosine to *P. aeruginosa* increased biofilm formation (53). Moreover, changes in *Staphylococcus aureus* pentose phosphate pathway have been found to affect biofilm formation and virulence (54). Therefore, the relationship between metabolism and resistance in NDM-5-producing CRKP is also worth exploring, which will be the focus of our future research.

Then, based on previous research, readings with a length of 50–500 nt and stable secondary structure, which unmatched with the reference gene and NR database, are listed as candidate sRNAs (55, 56). We mined a total of 268 candidate sRNAs, of which 3 were upregulated and 10 were downregulated in NDM-5-producing CRKP compared with CSKP. We subsequently matched known sRNAs by comparing candidate sRNAs with sRNAMap database and the Rfam database, which are often used to annotate known sRNAs in bacteria, and found 20 known sRNAs and 248 newly discovered sRNAs (57, 58). A study identified 475 new sRNAs in the *Salmonella enterica* of carbon starvation and demonstrated that one of the newly discovered sRNAs regulates the response of *Salmonella* to carbon starvation (59). In addition, using RNA-seq, our research group identified 112 novel sRNAs in the KPC-2-producing CRKP and found that sRNA51 could modulate its resistance by inhibiting the expression of *acrA* (22).

After we demonstrated that qRT-PCR results were consistent with RNA-seq results, we selected sRNA207 to further research. CPAT software result of sRNA207 showed that Fickett score <0.74, and the value of Hexamer is negative. Fickett score ≤0.74 indicates that RNA has no coding ability, and the Hexamer score value is negative, indicating that it is unlikely to encode protein (34, 35). Therefore, it is further proved that sRNA207 is a 136-nt sRNA with no coding capability. We subsequently predicted the target genes of sRNA207 by IntaRNA 2.0.3, including *yciG*, *ycgZ*, *glpR*, *smf-1*, *deoA*, and *lvG.* Miyakoshi et al. also used IntaRNA 2.0.3 to predict 21 target genes of sRNA GcvB in *E. coli* (49). Many studies have proved that *smf-1*, the gene encoding fimbriae in *Stenotrophomonas maltophilia*, is closely related to the formation of biofilm (37, 60). Through blast, we found that *smf-1* was also a fimbriae-related gene in *K. pneumoniae* (Table S7). Fimbriae are hair-like structures that extend from the surface of the organism and promote the adherence of *K. pneumoniae* to both biological and abiotic surfaces, forming biofilm and leading to infection (61, 62). Biofilms are thought to be a way for bacteria to protect themselves against harsh environments and are closely related to drug resistance. It has been proved that the antimicrobial resistance of bacterial biofilms maybe 10–1,000 times higher than that of planktonic bacteria (63, 64). The first step of biofilm formation is initial attachment, so fimbriae are essential for biofilm formation (65). Many fimbriae-related genes are involved in biofilm formation in *K. pneumoniae*, such as *fim* and *mrk* gene, which encode fimbriae 1 and 3, and more and more fimbriae genes have been discovered in recent years, such as *ecp* and *kpa* to *kpg* gene clusters (64, 65). And when *K. pneumoniae* grows as a biofilm, resistance to antimicrobial agents increases dramatically (38, 66). We discovered that NDM-5-producing CRKP has a stronger biofilm-forming ability than CSKP, and both RNA-seq (Table S3) and qRT-PCR (Fig. 3E) results showed higher *smf-1* expression levels in NDM-5-producing CRKP. To examine the effect of sRNA207 on *smf-1* expression levels and biofilm formation of NDM-5-producing CRKP, we knocked down sRNA207 in NDM-5-producing CRKP. The results showed that the expression of *smf-1* and biofilm formation ability of the sRNA207(−) strains were decreased, and more exciting was that the resistance of the sRNA207(−) strains to carbapenem antibiotics also significantly reduced. Actually, many studies have found that sRNA can regulate the formation of biofilm, which leads to bacterial resistance to antibiotics. For example, suppression of sRNA00203 in *Acinetobacter baumannii* impaired biofilm formation and sensitized the biofilm cells to imipenem and ciprofloxacin (67). The direct binding of ProQ to the sRNA RyfA was crucial for avian pathogenic *Escherichia coli* biofilm formation, pathogenicity, adhesion, and intracellular survival (68). Therefore, we hypothesized that sRNA207 could target binding of *smf-1* and stabilize its expression, thereby promoting biofilm formation, ultimately leading to increased resistance. To test this hypothesis, we knocked down *smf-1* in NDM-5-producing CRKP and found that biofilm formation of *smf-1*-knockdown strains was reduced (Fig. 4A and B), and compared with NDM-5-producing CRKP, the MIC of *smf-1*-knockdown strains decreased from 128 μg/mL to 16 μg/mL for meropenem, from 128 μg/mL to 32 μg/mL for ertapenem, and from 128 μg/mL to 32 μg/mL for imipenem. These results suggest that *smf-1* plays an influential role in the regulation of biofilm formation and antibiotic sensitivity in NDM-5-producing CRKP.

In this paper, we revealed the transcription and sRNA expression profiles of NDM-5-producing CRKP and proved that sRNA207 may promote the biofilm formation by stabilizing the expression of *smf-1*, thus affecting the level of carbapenem resistance of *Klebsiella pneumoniae.* This not only enriches our understanding of the transcriptome of NDM-5-producing CRKP and the development of drug resistance, but also provides a new therapeutic target for the clinic.

## MATERIALS AND METHODS

### Bacterial strains

The *K. pneumoniae* used in this study were from the strain bank of Shengjing Hospital affiliated to China Medical University, which contained a variety of clinical bacteria and fungi in the past 10 years. Eight strains of CSKP from patients with pneumonia and eight strains of NDM-5-producing CRKP from patients with septicemia were randomly selected for experiments using the SPSS random sampling method. And the strains were ST11.

### Antimicrobial susceptibility testing

The drug sensitivity test was conducted using the Phoenix-100(BD) compact system, and the results were interpreted according to the Clinical and Laboratory Standards Association interpretation standards.

Microbroth dilution method was used to detect the resistance of experimental strains (69, 70). Simply, the concentration of the bacterial solution was adjusted to 1 × 10^6^ CFU/mL, and the concentration of meropenem was prepared to 2,048 µg/mL using Muller-Hinton agar (MH) medium. The 50 µL MH was added to 1–10 holes of the 96-well plates, and 10 µL MH was added to 12 holes of the 96-well plates. Add 50 µL of the drug to the 11th and 10th holes, then mix the drug in the 10th hole and suck it out into the 9th hole, repeat the operation. When the drug is added to 2 well, mix the drug well, suck out 50 µL and discard it, and add 50 µL diluted bacterial solution to 1–11 well. The final drug concentration of meropenem was 1,024, 512, 256, 128, 64, 32, 16, 8, 4, and 2 µg/mL. The 96-well plates were cultured in a constant temperature incubator at 35°C for 16–18 h, and the growth was observed by the naked eye. The MIC was the lowest aseptic growth concentration. And the microbroth dilution methods of imipenem and ertapenem are consistent with meropenem.

### Extraction of RNA

NDM-5-producing CRKP and CSKP were cultured overnight under 37°C with shaking at 200 rpm. The total RNA was prepared using the SPINeasy RNA Kit for Bacteria (with Lysing Matrix) (MP).

### RNA-seq

RNA sequencing was performed on four strains of NDM-5-producing CRKP and four strains of CSKP using the SPSS random sampling method. Original data were obtained by Gene Denovo Biotechnology (Guangzhou, China) using Illumina Novaseq 6000. And we used quality control software fastp (https://github.com/opengene/fastp) to reduce data noise and get effective data. The retained reads were compared with the Kp genome assembly ASM2286966v1 using Bowtie2 (version 2.2.8), and the genes that could be aligned were considered as known genes.

Referring to the previous study (55, 56), we used Rockhopper to annotate fragments that did not match the reference genome. Unannotated genes with a length of 50–500 nt and stable secondary structure were listed as candidate sRNAs (57). In addition, we used the Rfam database (version 13) and sRNAMap database (version 2009) to annotate sRNAs.

### Analysis of RNAs and sRNAs

We used RSEM software to obtain the expression levels of RNAs and sRNAs by calculating the fragment map per million per kilobase transcript (71). In addition, the edgeR package (http://www.r-project.org/) was used to calculate the DEGs and differential expression sRNAs between samples with the criteria of FDR < 0.05 and |log_2_FC| > 1.

KEGG pathway enrichment analysis uses a hypergeometric detection method to identify the pathways of DEGs. GO analysis maps all differential genes to molecular function, biological process, and cellular components.

CPAT software was used to evaluate whether sRNAs had protein-coding functions (33). Moreover, IntaRNA 2.0.3 was used to predict the target mRNAs of sRNAs and the binding sites between them (drawn using FigDraw).

### qRT-PCR

According to the reverse transcription kit (Vazyme), the total RNA of 1 µg was used to prepare cDNA, and according to the instructions of 2× ChamQ Universal SYBR qPCR Master Kit (Vazyme), 20 µL of qPCR is carried out each time with 2 µL as a template. The primers are shown in Table S8. The *rpoB* gene was used as a reference gene for calculation of the relative target gene expression using the 2^−ΔΔCt^ method.

### Biofilm experiment

The bacteria were cultured overnight in a centrifuge tube with 5 mL Luria-Bertani (LB) medium in a shaker with 200 rpm at 37°C. The concentration of bacterial solution was adjusted to 1 × 10^7^ CFU/mL, and 100 µL was cultured in 96-well plates at 35°C for 24 h. Then the bacterial solution was removed, 120 µL sterile phosphate buffer saline ( PBS) was added to each well, gently washed three times, and dried at room temperature for 20 min. Add 1% crystal violet solution 100 µL to each well and stain for 20 min. Remove the crystal violet solution, rinse each well with 100 µL PBS until colorless, soak for 5 min each time, and air dry at room temperature for 20 min. Finally, 100 µL 95% ethanol was added to each well for decolorization for 1 h, and the optical density at 590 nm was measured.

### Construction of sRNA207 mutant and *smf* mutant

We commissioned KMD Biosciences to construct a plasmid containing the chloramphenicol resistance gene. First, the sRNA207 was amplified using forward primer TGCTGGTTTAATCGGTACCCGGGG and reverse primer GCCATTCTCCGGTCGACTCTAGAGGATC. Then the gene was cloned into the NotI and BamHI sites of pKO3-km, which contains the chloramphenicol resistance gene and electroporated into *E. coli* S17-1 λpir and confirmed by sequence analysis. The receptor NDM-5-producing CRKP and the donor *E. coli* were incubated overnight at 37°C, respectively, until the optical density at 600 nm reached 0.8. The mixture was mixed at a ratio of 2:1 (donor: acceptor), then spread on LB agar plates and incubated at 37°C overnight. Transconjugants were selected by plating on LB agar with chloramphenicol. Clones were screened by PCR to verify the loss of the targeted gene. The primers are shown in Table S8.

The *smf* mutant was generated by allelic exchange by using pKO3-km, a suicide vector that allows the use of chloramphenicol for selection. Sequences upstream and downstream (~1,000 bp each) were generated by PCR with the primer sets indicated in Table S8 and cloned into pKO3-km, and confirmed by sequence analysis. Subsequent operations are consistent with the above (72).

### Statistical analysis

Statistics were calculated using GraphPad Prism 9.0.0. Firstly, the Shapiro-Wilk method is used to test whether the calculated data accord with the normal distribution. The Mann-Whitney test was used if the data did not conform to a normal distribution. The variance was tested if it did; the unpaired *t*-test was used if the variance was consistent; and the Welch correction was used if the variance was inconsistent. When *P* < 0.05, the difference was considered to be statistically significant.

## Data Availability

The sequences are deposited in the NCBI Sequence Read Archive under BioProject ID PRJNA1006790.

## SUPPLEMENTAL MATERIAL

Table S1 spectrum.01537-24-s0001.xlsx Sources of Klebsiella pneumoniae.

Table S2 spectrum.01537-24-s0002.xlsx DEGs of NDM-5-producing CRKP.

Table S3 spectrum.01537-24-s0003.xlsx KEGG pathways of DEGs.

Table S4 spectrum.01537-24-s0004.xlsx GO of DEGs.

Table S5 spectrum.01537-24-s0005.xlsx sRNAs in NDM-5-producing CRKP and CSKP.

Table S6 spectrum.01537-24-s0006.xlsx Target mRNA of sRNA207.

Table S7 spectrum.01537-24-s0007.xlsx Blast of smf-1.

Table S8 spectrum.01537-24-s0008.xlsx Primer sequence.

ASM does not own the copyrights to Supplemental Material that may be linked to, or accessed through, an article. The authors have granted ASM a non-exclusive, world-wide license to publish the Supplemental Material files. Please contact the corresponding author directly for reuse.
